# Resveratrol regulates macrophage recruitment and M1 macrophage polarization and prevents corneal allograft rejection in rats

**DOI:** 10.3389/fmed.2023.1250914

**Published:** 2023-10-23

**Authors:** Chenjia Xu, Ruilin Guo, Chao Hou, Minglu Ma, Xiaojuan Dong, Chen Ouyang, Jing Wu, Ting Huang

**Affiliations:** State Key Laboratory of Ophthalmology, Zhongshan Ophthalmic Center, Sun Yat-sen University, Guangdong Provincial Key Laboratory of Ophthalmology and Visual Science, Guangzhou, China

**Keywords:** resveratrol, macrophages, corneal transplantation, immune rejection, PI3K/Akt pathway

## Abstract

**Introduction:**

Resveratrol is an immune modulator that can reduce M1 macrophage polarization *in vitro*. Reducing macrophage recruitment and M1 polarization can prevent corneal allograft rejection (CGR). In this study, rat corneal allograft rejection models were established to explore the effects of resveratrol on CGR and macrophages and the underlying mechanisms after corneal transplantation.

**Methods:**

Corneal allograft models were established, and 100 mg/kg resveratrol was injected intraperitoneally. The corneal allografts were assessed clinically using the Holland rejection scoring system, anterior segment photography, and anterior segment optical coherence tomography. Corneal macrophages, pro-inflammatory cytokines, and corneal lymphatic vessels were detected using hematoxylin and eosin staining, immunofluorescence staining, and real-time quantitative polymerase chain reaction (qRT-PCR). Dendritic cells (DCs) in cervical lymph nodes were explored using flow cytometry. RNA sequencing experiments were conducted to identify the mechanisms through which resveratrol affected CGR. The results were verified using Simple Western analysis. Pro-inflammatory cytokines by macrophages *in vitro* were measured using qRT-PCR and enzyme-linked immunosorbent assays.

**Results:**

Resveratrol significantly prolonged the survival of corneal grafts and reduced graft edema and central corneal thickness. Corneal macrophage recruitment and M1 macrophage polarization decreased significantly after corneal transplantation in the resveratrol group. Resveratrol also reduced pro-inflammatory cytokines in corneal grafts and suppressed the early generation of cornea lymphatic vessels and the recruitment of cornea inflammatory cells 14 days after surgery. Resveratrol decreased the proportion of DCs in ipsilateral cervical lymph nodes. The effect of resveratrol on CGR was related to the phosphatidylinositol 3-kinase/protein kinase-B (PI3K/Akt) pathway. Resveratrol reduced the secretion of pro-inflammatory cytokines by M1 macrophages *in vitro*.

**Conclusion:**

Our findings suggest that resveratrol can reduce corneal macrophage recruitment and M1 macrophage polarization after corneal transplantation in rats and prevent CGR. The PI3K/Akt pathway may be an important mechanism that warrants further research.

## Introduction

Corneal opacity is an important blinding eye condition worldwide. Corneal transplantation surgery remains the main treatment for corneal opacity and has solved most of the blindness caused by corneal opacity (1, 2). The cornea has the highest transplantation success rate of all human organs because of its avascular nature and anterior chamber-associated immune deviation (3, 4). However, corneal allograft rejection (CGR) remains the major reason for the failure of penetrating keratoplasty (5). Although many drugs targeting CGR, such as corticosteroids, tacrolimus, and cyclosporine, play important roles in resisting CGR, they are mostly immunosuppressants, and long-term use may cause many side effects (6, 7). Therefore, there is an urgent need to find new drugs that can prevent or delay CGR.

Macrophages originate from monocytes and play a key role in innate immunity, inflammatory responses, and tissue homeostasis (8). Low expression levels of major histocompatibility complex II (MHC II) co-stimulatory molecules in macrophages in eye tissues enable macrophages to act as antigen-presenting cells (APCs) in CGR (9). After corneal transplantation, the number and function of dendritic cells (DCs), cytotoxic T lymphocyte activity, and corneal neovascularization may decrease with a decrease in the number of macrophages, thereby delaying CGR (10–12). Allograft transplantation leads to the release of many chemicals, triggering the activation of macrophages. Depending on the surrounding inflammatory environment, macrophages can polarize into pro-inflammatory (M1) or anti-inflammatory (M2) macrophages. M1 and M2 macrophages can be derived from M0 macrophages *in vitro* due to different stimuli (13). M1 macrophages can be stimulated, among others, by lipopolysaccharides (LPS) and interferon gamma and can secrete pro-inflammatory cytokines, such as interleukin-6 (IL-6), IL-1β, and tumor necrosis factor alpha (TNF-α) (14, 15). IL-4 can polarize M0 into M2 macrophages, which can secrete anti-inflammatory cytokines, such as IL-10 (15). In mouse corneal transplant rejection models, M1 macrophages have been found to infiltrate the cornea in large numbers, suggesting that they may be directly involved in CGR (16). Previous studies have also shown that inhibiting M1 macrophage polarization can delay CGR (17, 18).

Pathological lymphatic vessels help transport APCs from the transplant site to the corresponding drainage area of lymphatic tissue, thereby accelerating CGR (19). Therefore, inhibiting corneal lymphatic vessels may be a viable strategy for preventing CGR. Researchers have pre-cultured corneal donor tissue *in vitro* and used anti-vascular endothelial growth factor (VEGF) drugs to block postoperative angiogenesis and lymphangiogenesis and maintain the transparency of corneal grafts. This can result in a significant decrease in corneal macrophages (20). Moreover, the inhibition of corneal lymphatic vessel formation can reduce the incidence of corneal transplant rejection, and lymphangiogenesis is associated with macrophages (21).

Resveratrol is a non-flavonoid polyphenol compound found in fruits such as blueberries and many red grape varieties (22, 23). Resveratrol plays an important role in various biological functions, exerting anti-inflammatory, antioxidant, anticancer, and anti-neurodegenerative effects (24–26). A previous study found that resveratrol mainly had anti-inflammatory effects on macrophages (27). Other studies have shown that it can prolong the survival of liver allografts (28, 29). In summary, resveratrol may be a relatively safe drug that can prevent CGR and may act by affecting macrophages. This study aimed to examine whether resveratrol can affect CGR by interfering with the recruitment and polarization of corneal macrophages.

## Materials and methods

### Animals

Six-to-eight-weeks-old female Sprague Dawley (SD) rats and Wistar rats were purchased from Guangzhou Southern Medical University Experimental Animal Technology Development Co., Ltd. (Guangzhou, Guangdong, China) To avoid the interference of sex hormones with immune responses, only female rats were used. The rats were housed in a specific pathogen free (SPF)-grade standardized feeding room at Zhongshan Ophthalmic Center, Yat-sen University. The experiments were approved by the Institutional Animal Care and Use Committee of Zhongshan Ophthalmic Center (protocol code O2021044).

### Corneal transplantation model

Preoperatively, pentobarbital sodium (1%) was used as an anesthetic in intraperitoneal injection doses of 40–45 mg/kg. To establish allograft corneal transplantation models, the SD rats were used as donors, and the Wistar rats were used as recipients. Moreover, corneal autograft operations were performed on Wistar rats. Full-thickness corneal donors 3.5 mm in diameter were sutured to implant beds 3.0 mm in diameter using 10-0 nylon sutures (Alcon, Fort Worth, TX, United States) under a microscope.

Four experimental groups were formed. A normal group of Wistar rats did not undergo corneal transplantation and received no treatment. The Wistar rats in the autograft group underwent only autologous corneal transplantation and received no treatment. A control group of Wistar rats underwent corneal allograft surgery and received saline treatment. The resveratrol group consisted of Wistar rats undergoing corneal allograft surgery and receiving resveratrol treatment. Resveratrol solution (0.6 mL, 100 mg/kg, Sigma-Aldrich, St. Louis, MO, United States) or saline was injected intraperitoneally every day for 30 days postoperatively.

### Cell treatments

A human monocytic leukemia cell line (THP-1) was obtained from the cell bank of the Chinese Academy of Sciences in Shanghai. An RPMI-1640 culture medium (Gibco-BRL, Grand Island, NY, United States) with 10% FBS (Thermo Fisher Scientific, Waltham, MA, United States) and 1% penicillin-streptomycin (Sigma-Aldrich, St. Louis, MO, United States) was used. All cells were cultured in a cell incubator containing 5% carbon dioxide at 37°C. After cell line resuscitation, fourth-to-eighth-generation cells were used in the experiments. Adhesive THP-1 cells induced using phorbol 12-myristate-13-acetate (PMA; 100 ng/mL, P1585; Sigma-Aldrich, St. Louis, MO, United States) for 48 h were considered M0 macrophages. M0 macrophages were transformed into M1 macrophages using LPS treatment (1 μg/mL, from Escherichia coli O111:B4, Sigma-Aldrich, St. Louis, MO, United States) for 24 h.

The cells were divided into five groups. The control group consisted of untreated M0 macrophages. The cells in the LPS group were treated only with LPS. The cells in the resveratrol groups were treated with three resveratrol concentrations: 10, 25, and 50 μM.

### Clinical assessment

The rat corneas in the autograft, control, and resveratrol groups were examined using slit-lamp microscopy every other day for 30 days postoperatively. The corneal allografts were assessed clinically using the Holland rejection scoring system (30, 31). Corneal graft clarity, edema, and neovascularization were used as indicators (Table 1). The three parameters were summed to obtain a rejection index (RI). An RI of ≥6 was considered to indicate rejection. Anterior segment photography was performed 7 and 14 days postoperatively to record the condition of the corneal grafts in the control and resveratrol groups. Anterior segment optical coherence tomography (AS-OCT) was performed 14 days postoperatively to measure central corneal thickness (CCT) in the control, resveratrol, and normal groups.

**Table 1:** Standards of grading for corneal grafts.

Standard	Score	Sign
Clarity	0	Clear cornea
	1	Slight haze
	2	Increased haze but anterior chamber structures still clear
	3	Advanced haze with difficult view of anterior chamber
	4	Opaque cornea without view of anterior chamber
Edema	0	No stromal or epithelial edema
	1	Slight stromal thickness
	2	Diffuse stromal edema
	3	Diffuse stromal edema with microcystic edema of epithelium
	4	Bullous keratopathy
Neovascularization	0	No vascularization
	1	Vascularization of the peripheral cornea
	2	Vascularization to the corneal wound
	3	Vascularization of the peripheral graft
	4	Vascularization of the entire graft

The grafts showing a score of RI ≥6 were defined as rejected.

### Hematoxylin and eosin staining

On the 14th postoperative day, the eyeballs of rats in the normal, control, and resveratrol groups were removed and placed in 4% paraformaldehyde (PFA; Biosharp, Hefei, Anhui, China) solution at 4°C overnight. After dehydration and paraffin embedding, sections approximately 4 μm thick were cut. After dewaxing and hydration, the sections were stained with hematoxylin and eosin (H&E; Servicebio, Wuhan, Hubei, China). Histological analysis was performed using an inverted microscope (Ts2FL; Nikon, Japan) and CapStudio software.

### Immunofluorescence staining

The eyes of rats in the normal, control, and resveratrol groups were removed and fixed with 4% PFA. The tissues were then embedded in paraffin wax and cut into 4 μm thick sections. The sections were stained with mouse anti-cluster of differentiation molecule 11b (CD11b; 1:100, sc-1186; Santa Cruz, CA, United States) and rabbit anti-inducible nitric oxide synthase (iNOS; 1:500, ab283655; Abcam, Cambridge, United Kingdom) primary antibodies at 4°C overnight. Subsequently, they were dyed with Goat anti-Mouse IgG (H + L) Highly Cross-Adsorbed Secondary Antibody (Alexa Fluor Plus 488, 1:200; Invitrogen, Carlsbad, CA, United States) and Goat anti-Rabbit IgG (H + L) Highly Cross-Adsorbed Secondary Antibody (Alexa Fluor Plus 594, 1:200; Invitrogen) for 1 h. The stained slices were analyzed under a Zeiss LSM 880 microscope (Carl Zeiss Meditec, Jena, Germany).

### Real-time quantitative polymerase chain reaction

On the seventh postoperative day, corneas from the normal, control, and resveratrol groups were excised and cut into pieces. An RNeasy Fibrous Tissue Mini Kit (Qiagen, Duesseldorf, Germany) was used to extract RNA from the corneas. An RNA Quick Purification Kit (ESscience, Shanghai, China) was used to extract RNA from macrophages. The RNA was then reverse-translated into cDNA using HiScript II QRT SuperMix (Vazyme, Nanjing, Jiangsu, China). A LightCycler 480 SYBR Green I Master (Roche, Basel, Switzerland) was used to perform a real-time quantitative polymerase chain reaction (qRT-PCR). The primers are shown in Tables 2, 3.

**Table 2:** The primer sequences of the rat genes.

Gene	Primer sequence (5′–3′)
GAPDH	
Forward	TCTCTGCTCCTCCCTGTTC
Reverse	ACACCGACCTTCACCATCT
TNF-α	
Forward	TACTGAACTTCGGGGTGATTGGTCC
Reverse	CAGCCTTGTCCCTTGAAGAGAACC
IL-1β	
Forward	CCCTGCAGCTGGAGAGTGTGG
Reverse	TGTGCTCTGCTTGAGAGGTGCT
IL-6	
Forward	CGAAAGTCAACTCCATCTGCC
Reverse	GGCAACTGGCTGGAAGTCTCT
MCP-1	
Forward	CTGGAGAACTACAAGAGAAT
Reverse	TCTAGTATTCATGGAAGGGA
iNOS	
Forward	CACGACACCCTTCACCACAAG
Reverse	TTGAGGCAGAAGCTCCTCCA
VEGF-C	
Forward	GATTCAGGGGTTGATTTCTTG
Reverse	TTTCCTTAATTCATGTGGAGCC

**Table 3:** The primer sequences of the human genes.

Gene	Primer sequence (5′–3′)
GAPDH	
Forward	GGAGTCCACTGGCGTCTTCA
Reverse	GTCATGAGTCCTTCCACGATACC
TNF-α	
Forward	GAAAGCATGATCCGGGACGTG
Reverse	GATGGCAGAGAGGAGGTTGAC
IL-1β	
Forward	ATGGCTTATTACAGTGGCAATGAG
Reverse	GTAGTGGTGGTCGGAGATTCG
IL-6	
Forward	ACTCACCTCTTCAGAACGAATTG
Reverse	CCATCTTTGGAAGGTTCAGGTTG
MCP-1	
Forward	GATCTCAGTGCAGAGGCTCG
Reverse	TGCTTGTCCAGGTGGTCCAT
VEGF-C	
Forward	CAGCACGAGCTACCTCAGCAAG
Reverse	TTTAGACATGCATCGGCAGGAA

### Whole-mount corneal immunofluorescence

On the seventh postoperative day, whole corneas from the control and resveratrol groups were excised and fixed with 4% PFA for 1 h. The corneas were then digested by protease K and permeated with methanol. Subsequently, they were blocked at 4°C overnight with 10% Bovine Serum Albumin (BSA; Epizyme, Shanghai, China) blocking solution containing 0.5% Triton X-100. The corneas were then dyed with rabbit anti-rat lymphatic vessel endothelial hyaluronan receptor 1 (LYVE-1; 1:200, 11-036; AngioBio, San Diego, CA, United States) at 4°C overnight. Finally, they were stained with Goat anti-Rabbit IgG (H + L) Highly Cross-Adsorbed Secondary Antibody (Alexa Fluor Plus 594, 1:200, Invitrogen, Carlsbad, CA, United States) at room temperature for 2 h.

### Flow cytometry

On the 10th postoperative day, the right cervical lymph nodes on the same side as the surgical eyes of the rats in the normal, control, and resveratrol groups were removed and ground. The obtained cells were resuspended using stain buffer and surface-stained with CD103 (Integrin alpha E) Monoclonal Antibody (OX62), PE, eBioscience (12-1030-82, 0.25 μg per test; Thermo Fisher Scientific, Waltham, MA, United States) for 20 min. Analyses were then performed using a BD LSRFortessa flow cytometer (BD Biosciences, San Jose, CA, United States).

### RNA sequencing

Total RNA was extracted from corneas in the control and resveratrol groups using the method described above. A NanoDrop 2000 spectrophotometer (Thermo Fisher Scientific, Waltham, MA, United States) was used to evaluate the purity and quantification of the extracted RNA. The libraries were sequenced on a NovaSeq 6000 platform (Illumina, San Diego, CA, United States). DESeq25 (Siemens, Berlin, Germany) was used to analyze differential expression. *Q*-values of <0.05 were considered to indicate significant differences in differentially expressed genes (DEGs). R (v 3.2.0) was used to perform the gene ontology (GO) 6 and Kyoto encyclopedia of genes and genomes (KEGG) 7 pathways of DEGs to identify significant enrichment terms and draw a histogram based on the hypergeometric distribution. All RNA sequencing and analyses were performed by Ouyi Biomedical Technology Co., Ltd. (Shanghai, China).

### Simple western analysis

On the seventh postoperative day, proteins were isolated from corneas in the control and resveratrol groups. After measuring protein concentrations using the bicinchoninic acid assay (BCA) method, the samples were prepared and denatured at 95°C for 5 min. Following the protocol described in the manual for the 12–230 kDa Wes Separation Module (SM-W004; ProteinSimple, Bio-Techne, San Jose, CA, United States), an automated capillary electrophoresis system was used to separate and detect the proteins. Antibodies targeting the proteins GAPDH, PI3K, Akt and Phospho-Akt (p-Akt) were used [GAPDH (1:50, 5174S; Cell Signaling Technology, Danvers, MA, United States), PI3K (1:50, 4249S; Cell Signaling Technology, Danvers, MA, United States), AKT (1:600, 4691S; Cell Signaling Technology, Danvers, MA, United States), and p-Akt (1:10, 4060S; Cell Signaling Technology, Danvers, MA, United States)]. Horseradish peroxidase (HRP)-conjugated secondary anti-rabbit antibody was used to detect signals. Compass software (ProteinSimple) was used for the quantitative analysis.

### Enzyme-linked immunosorbent assay

The cell supernatants of the five groups of cells were extracted separately. Cytokines secreted by macrophages were measured using enzyme-linked immunosorbent assays (ELISA; TNF-α ELISA Kit BMS2034, IL-6 ELISA Kit BMS213-2, MCP-1 ELISA Kit BMS281INST, and VEGF-C ELISA Kit BMS297-2; Thermo Fisher Scientific, Waltham, MA, United States).

### Statistical analysis

The data were analyzed using GraphPad Prism 7 software. Kaplan–Meier survival curves were drawn to analyze corneal graft survival. Differences between the two groups were assessed using an independent-samples *t*-test. *p*-values of <0.05 were considered statistically significant.

## Results

### Resveratrol prolonged corneal allograft survival

**Figure 1:**
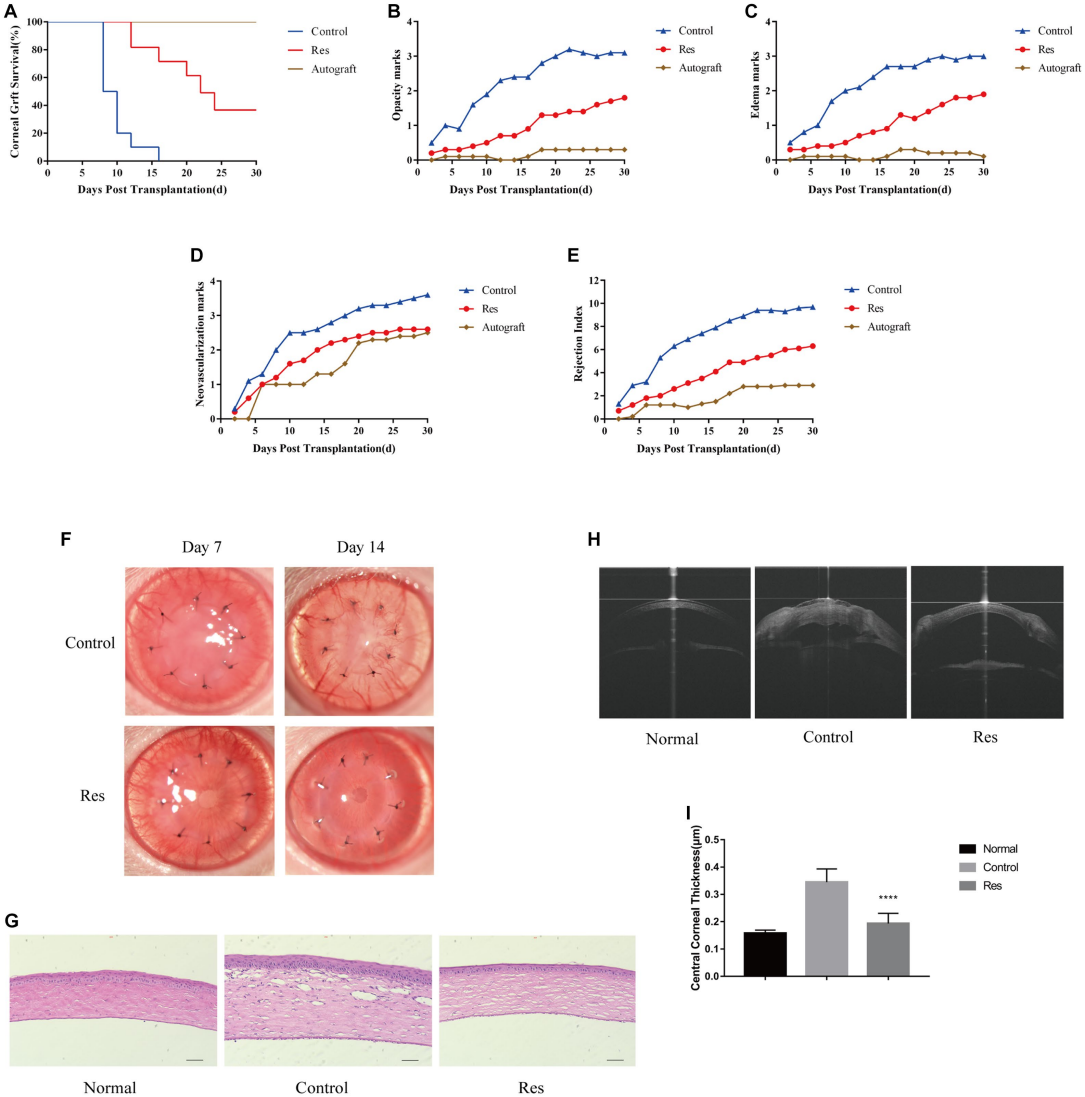
Resveratrol prevented CGR and inflammatory responses in rats. **(A)** Kaplan–Meier corneal allograft survival curve (*n* = 10). **(B–E)** The average clarity, edema, neovascularization, and RI scores were considerably lower in the resveratrol group than in the control group. **(F)** Anterior segment photographs of rats in the resveratrol and control groups on postoperative days 7 and 14. **(G)** Comparison of H&E staining images and inflammatory cell expression in the corneas of the normal, control, and resveratrol groups on postoperative day 14 (*n* = 3, magnification: 200×, scale bar: 50 μm). **(H,I)** AS-OCT images of corneas in the normal, control, and resveratrol groups on postoperative day 14. The corneas in the resveratrol group were significantly thinner than those in the control group. The data are shown as mean ± SD. ^****^p < 0.0001 between the resveratrol and control groups.

Graft survival time curves were plotted based on three scores, including corneal graft clarity, edema, and neovascularization (Figure 1). At the end of the 30 days observation period, all corneal grafts in the autograft group were still alive. In the control group, all grafts were rejected between days 8 and 12. The median survival time (MST) was 9 days. In the resveratrol group, six grafts were rejected between days 10 and 24. The MST was 17 days. The other four grafts in this group survived until the end of the observation period (Figure 1A). During the observation period, the average corneal graft clarity, edema, and corneal neovascularization scores in the resveratrol group were significantly lower than those in the control group (Figures 1B–E).

### Resveratrol reduced postoperative corneal inflammatory responses

Most corneal grafts in the control group showed inflammatory responses soon after surgery, which gradually worsened. In this group, neovascularization reached the periphery of almost all grafts around 7 days after surgery. In contrast, in the resveratrol group, neovascularization grew to the edge of most grafts on the 14th postoperative day. In this group, the grafts remained relatively transparent, with no significant edema and significantly milder rejection reactions (Figure 1F). On the 14th day after surgery, the corneas were used for histological analysis. The H&E staining results showed that inflammatory cells were significantly infiltrated in the corneas of rats with CGR. However, in the allografts of the resveratrol group, inflammatory cell infiltration was significantly decreased (Figure 1G).

### Graft CCT

Corneal thickness after corneal transplantation is related to transplant rejection. The average CCT in the control group was 0.345 ± 0.015 mm, whereas the average CCT in the resveratrol group was 0.194 ± 0.012 mm. (Normal rat CCT is 0.158 ± 0.004 mm.) The corneal grafts in the resveratrol group were significantly thinner than those in the control group (*p* < 0.01) (Figures 1H,I).

### Effects of resveratrol on macrophages in corneal grafts

**Figure 2:**
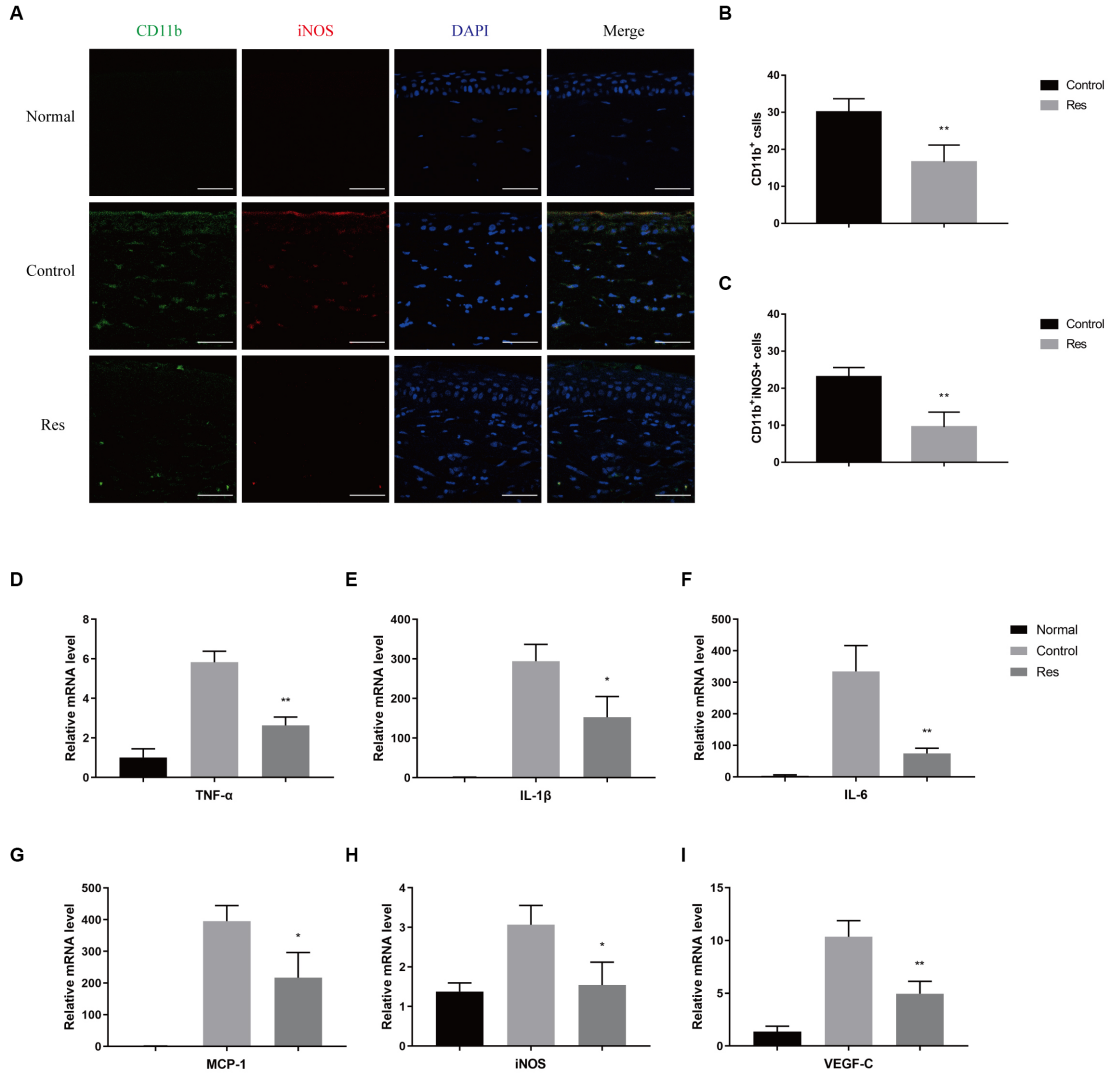
Changes in corneal macrophage recruitment and M1 macrophage polarization after resveratrol treatment in rats. **(A–C)** The resveratrol group had significantly lower numbers of CD11b^+^-labeled macrophages (green) and CD11b^+^ iNOS^+^-labeled M1 macrophages (red) than the control group on postoperative day 7 (*n* = 4, magnification: 400×, scale bar: 50 μm). **(D–I)** The resveratrol group had significantly lower mRNA expression levels of TNF-α, IL-1β, IL-6, MCP-1, iNOS, and VEGF-C than the control group on postoperative day 7 (*n* = 3). The data are shown as mean ± SD. ^*^p < 0.05 and ^**^p < 0.01 between the resveratrol and the control groups.

To investigate the recruitment and polarization of macrophages in corneas, macrophages were labeled with CD11b, and M1 macrophages were labeled with iNOS on the seventh postoperative day (Figure 2A). No macrophage infiltration was observed in normal corneal tissue. In the control group, the average number of CD11b^+^-labeled macrophages was 30 cells per field, and the proportion of CD11b^+^ iNOS^+^-labeled M1 macrophages was approximately 76.67% (Figure 2B). In the resveratrol group, the average number of CD11b^+^-labeled macrophages was 17 cells per field, and the proportion of CD11b^+^ iNOS^+^-labeled M1 macrophages was approximately 58.82% (Figure 2C). Resveratrol treatment resulted in significantly lower of CD11b^+^-labeled macrophage recruitment (*p* < 0.01) and a significantly lower polarization ratio of CD11b^+^ iNOS^+^-labeled M1 macrophages (*p* < 0.01) than saline treatment (Figures 2B,C). M1 macrophages secreted multiple types of pro-inflammatory cytokines, including TNF-α, iNOS, IL-1β, monocyte chemoattractant protein-1 (MCP-1), IL-6, and VEGF-C. The mRNA expressions of TNF-α, iNOS, IL-1β, MCP-1, IL-6, and VEGF-C were significantly lower in the resveratrol group than in the control group (Figures 2D–I).

### Resveratrol inhibited corneal lymphangiogenesis

**Figure 3:**
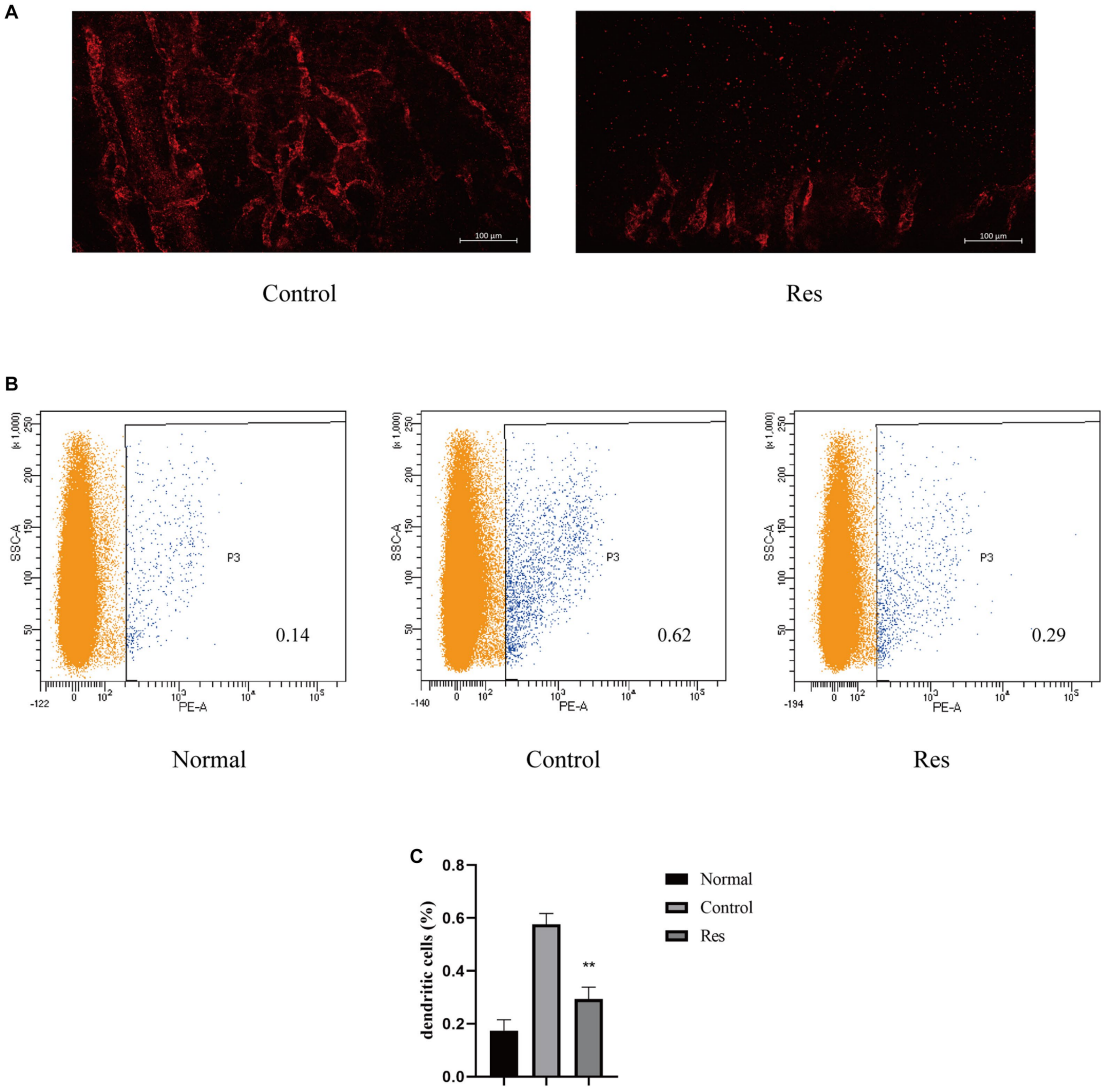
Resveratrol suppressed corneal lymphangiogenesis and the migration of DCs to the cervical lymph nodes after corneal transplantation. **(A)** Corneal lymphatic vessels stained with LYVE-1 (red). Resveratrol significantly suppressed corneal lymphangiogenesis 7 days after surgery (*n* = 3, scale bar: 100 μm). **(B,C)** The number and proportion of OX62-labeled DCs in the ipsilateral lymph nodes of rat corneal grafts were significantly lower in the resveratrol group than in the control group (*n* = 3). The data are shown as mean ± SD. ^**^p < 0.01 between the resveratrol and control groups.

Previous PCR-based studies have shown that resveratrol can reduce inflammatory cytokines related to the formation of corneal lymphatic vessels, including TNF-α, IL-1β, and VEGF-C. Seven days after surgery, the rats’ corneas were removed, and LYVE-1 was used as a lymphatic-specific marker for whole-mount corneal immunofluorescence to clarify the influence of resveratrol on the formation of new lymphatic vessels after corneal transplantation. In the control group, LYVE-1-labeled lymphatic vessels grew considerably from the corneal limbus to the grafts. In contrast, lymphatic vessels grew only slightly in the resveratrol group (Figure 3A).

### Transfer of DCs to cervical lymph nodes

After corneal transplant rejection, DCs, a main type of APCs, can be transported to the local lymph nodes on the same side through lymphatic vessels (32). The results reported above confirmed that resveratrol reduced the generation of corneal lymphatic vessels. The changes in APCs in cervical lymph nodes were further investigated using flow cytometry. DCs from the ipsilateral lymph nodes from the different treatment groups were labeled with OX62 (Figure 3B). The results showed a significant increase in the proportion of DCs in the cervical lymph nodes after corneal transplantation. However, on the 10th day after surgery, the resveratrol group showed significantly fewer DCs than the control group (Figure 3C).

### RNA sequencing results

**Figure 4:**
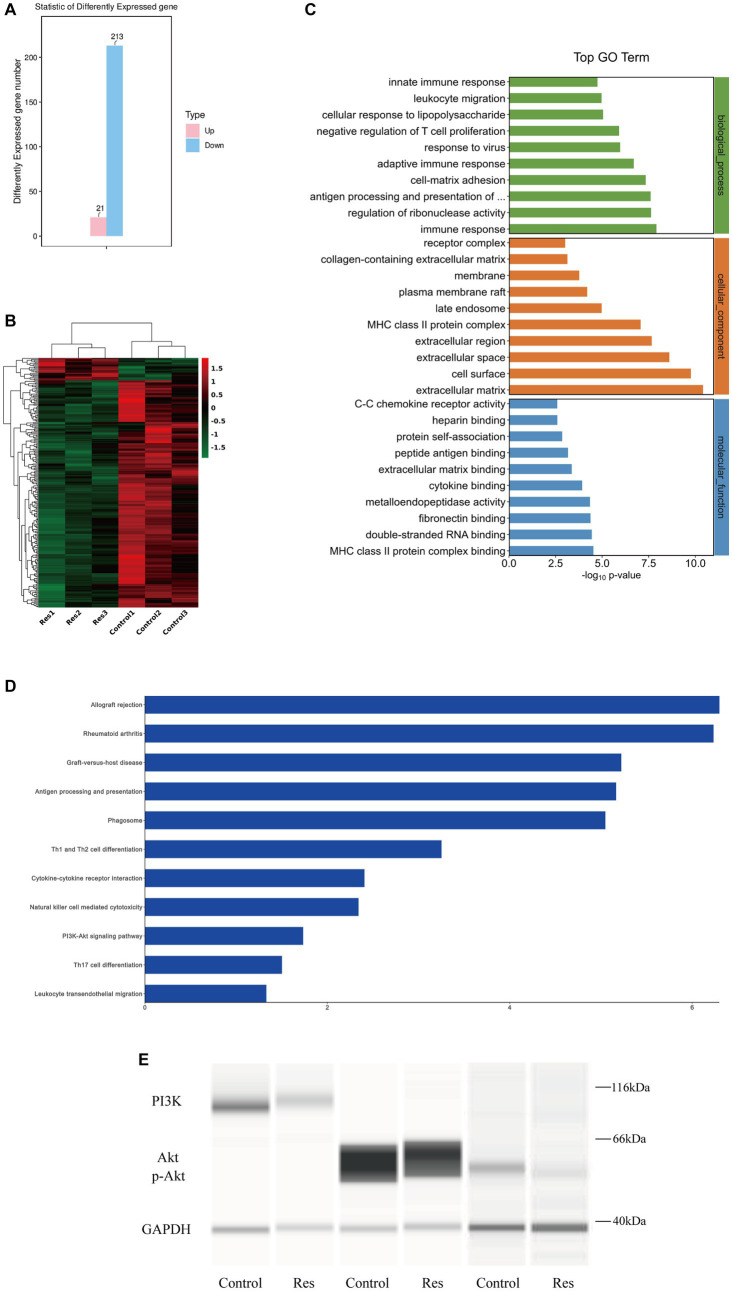
Results of RNA sequencing and protein verification of the PI3K/Akt pathway in the control and resveratrol groups. **(A)** Numbers of upregulated and downregulated DEGs in the two groups. **(B)** Differential gene expression heat map, with red indicating relatively high expression and green indicating relatively low expression of protein-coding genes. **(C)** Significant GO term enrichment statistics. The lower the GO term in the same category, the more meaningful it is. **(D)** KEGG pathway enrichment analysis. The higher the ranking, the more significant the change in the pathway. **(E)** Changes in PI3K/Akt signaling pathway protein expression after resveratrol treatment compared to the control group.

Total RNA was extracted from three corneas from the control group and three corneas from the resveratrol group. RNA transcriptome sequencing was performed, and the data were analyzed. A total of 234 DEGs, including 21 upregulated and 213 downregulated genes, were detected (Figure 4A). A differential gene expression heat map was created to visualize the grouping and clustering of DEGs (Figure 4B). The GO analysis showed that among the DEGs in the resveratrol group, immune response (biological process), extracellular matrix (cellular component), and MHC class II protein complex connection (molecular function) exhibited the greatest enrichment (Figure 4C). These results provided references for further research on related signaling pathways, and DEGs were analyzed for this purpose. The results showed that the downregulated genes in the resveratrol group regulated a total of 201 signaling pathways, of which 50 showed significant differences. Notably, in the KEGG analysis, one of the most important pathways of downregulated DEG enrichment in the resveratrol group was allograft rejection, suggesting that resveratrol may act against CGR (Figure 4D). Moreover, antigen processing and presentation of inflammatory-related diseases, Th1 and Th2 cell differentiation, Th17 cell differentiation, PI3K-Akt signaling pathway, and other related pathways were significantly enriched in the downregulation pathway of the resveratrol group. These results suggest that resveratrol may interfere with CGR through multiple pathways, indicating possible directions for follow-up research.

### Resveratrol inhibited PI3K/Akt pathway

Based on the RNA sequencing results reported above, the PI3K/Akt pathway was selected for protein-level pathway validation. The expressions of the PI3K and Akt proteins in the corneas of the rats in the control and resveratrol groups were analyzed using Simple Western. The protein table indicated that the expression levels of PI3K, Akt and p-Akt were significantly lower in the resveratrol group than in the control group (Figure 4E). These results suggest that resveratrol can effectively regulate the PI3K/Akt signaling pathway after corneal transplantation in rats, which may be related to its action against CGR.

### Resveratrol inhibited the polarization of M1 macrophages *in vitro*

**Figure 5:**
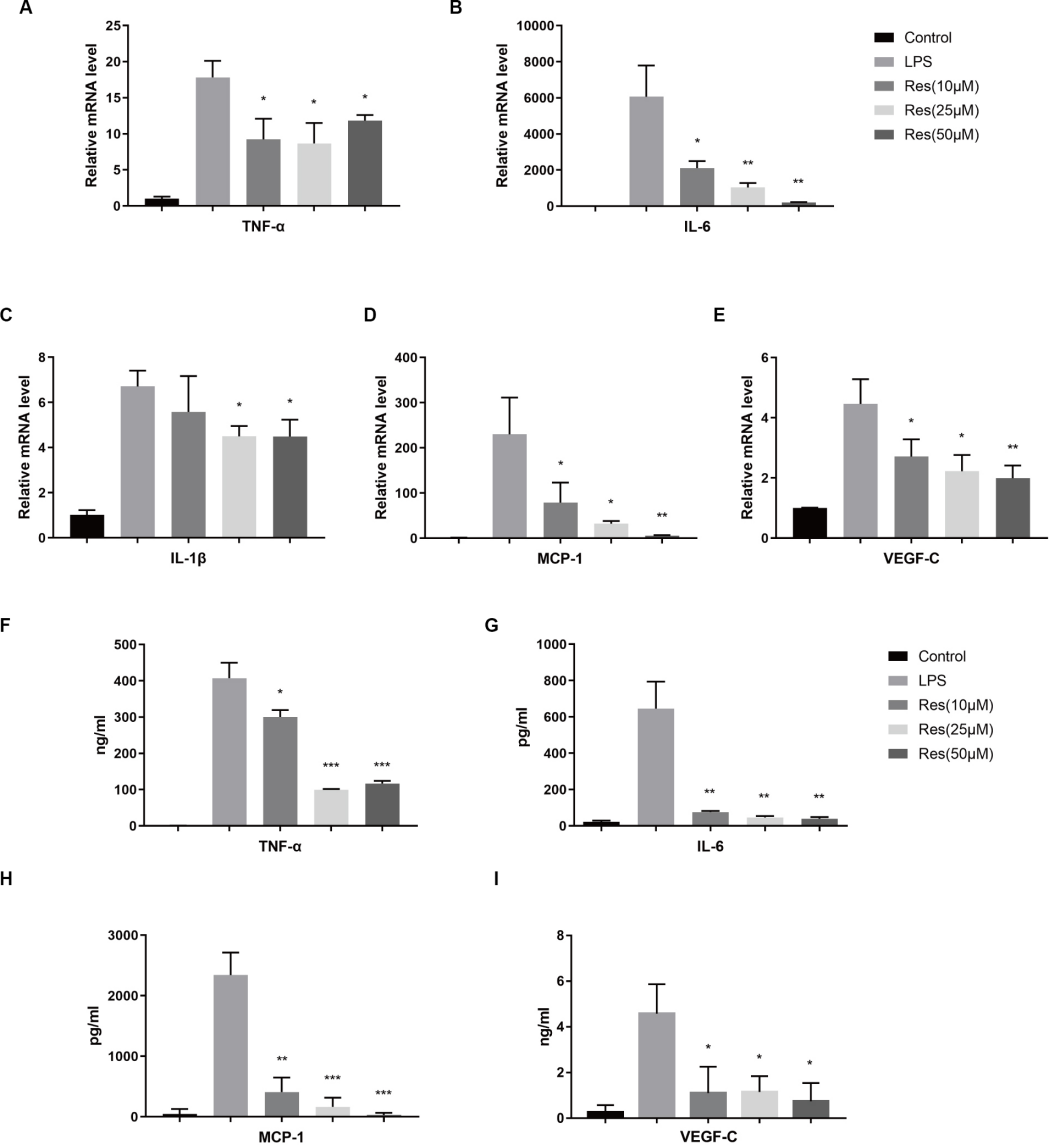
Effect of resveratrol on human macrophages *in vitro*. **(A–E)** Transcriptions of TNF-α, IL-6, IL-1β, MCP-1, and VEGF-C in different cell groups after LPS stimulation and resveratrol treatment. **(F–I)** Secretion of TNF-α, IL-6, MCP-1, and VEGF-C proteins in different cell groups after LPS stimulation and resveratrol treatment. The data are shown as mean ± SD. ^*^p < 0.05, ^**^p < 0.01, and ^***^p < 0.001 between the resveratrol and LPS groups.

THP-1 was used to validate the effect of resveratrol on macrophages *in vitro*. After being treated with PMA and LPS, THP-1 cells can be considered M1 macrophages, which express various pro-inflammatory cytokines. The qRT-PCR results showed that resveratrol at concentrations of 10, 25, and 50 μM reduced the production of pro-inflammatory cytokines, such as TNF-α, IL-6, IL-1β, MCP-1, and VEGF-C, by M1 macrophages (Figures 5A–E). The subsequent ELISA results were consistent with the qRT-PCR results (Figures 5F–I). Therefore, it can be concluded that resveratrol can inhibit the polarization of human M1 macrophages *in vitro*.

## Discussion

Resveratrol has an immunomodulatory effect and has been proven to affect transplant rejection. Wu et al. (29) found that resveratrol enhanced the inhibitory effect of cyclosporine on allogeneic liver rejection. The following studies showed that daily intraperitoneal injections of resveratrol (100 mg/kg) after liver transplantation significantly prolonged graft survival in rats (28, 33). Moreover, an *in vitro* study found that resveratrol reduced peripheral blood monocyte proliferation and stimulated T cell activation (34). However, the effect of resveratrol on CGR has hitherto been unclear. The results of this study showed that intraperitoneal injections of 100 mg/kg resveratrol significantly prolonged corneal graft survival and reduced inflammatory responses in rats.

Macrophages are inflammatory cells that play a crucial role in the occurrence of allogeneic rejection (35, 36). Early macrophage activation can mediate the activation of APCs, which is important for initiating immune rejection (37). Previous studies have shown that pro-inflammatory M1 macrophages promote CGR, while anti-inflammatory M2 macrophages can delay it to some extent (38, 39). Thus, inhibiting macrophage recruitment and M1 macrophage polarization can prevent CGR (16, 17). Resveratrol can regulate a wide range of inflammatory components and plays an immunomodulatory role in immune cells. Its anti-inflammatory activity mainly targets macrophages (27). In this study, resveratrol reduced the recruitment of CD11b^+^-labeled macrophages and the polarization ratio of CD11b^+^ iNOS^+^-labeled M1 macrophages compared to saline treatment. These results suggest that resveratrol can reduce macrophage recruitment and M1 macrophage polarization after corneal transplantation in rats, which may be a major reason for its action against CGR.

Previous studies indicated that resveratrol can inhibit the secretion of pro-inflammatory cytokines, such as IL-1, IL-6, and TNF-α, and increase the opposite effect of IL-10 produced by mouse macrophages, thereby regulating the activity of T cells and B cells (40, 41). M1 macrophages can secrete iNOS, IL-6, IL-1β, and TNF-α, which are pro-inflammatory cytokines associated with allograft rejection (39, 42). MCP-1, another important pro-inflammatory cytokine, can be secreted by macrophages during inflammation, significantly impacting the migration and infiltration of macrophages (43–45). Our experimental results are in line with previous findings. Resveratrol significantly reduced the mRNA expression levels of TNF-α, IL-1β, IL-6, MCP-1, iNOS, and VEGF-C in corneas in the early stages after corneal transplantation. This was mainly related to a reduction in pro-inflammatory macrophages. A reduction in pro-inflammatory cytokines may also affect the subsequent recruitment of inflammatory cells and prevent CGR in the early postoperative period. These results suggest that resveratrol can prevent CGR by inhibiting macrophage recruitment and M1 macrophage polarization. Our *in vitro* cell experiments also showed that resveratrol significantly reduced the secretion of various pro-inflammatory cytokines by M1 macrophages stimulated by LPS. The results indicated that resveratrol can suppress M1 macrophage polarization *in vitro*.

Pathological corneal lymphangiogenesis significantly increases CGR rates (46). It has been reported that lymphatic vessel formation is a more important risk factor for CGR than neovascularization (47, 48). The generation of corneal lymphatic vessels after corneal damage depends on macrophages (49). An animal study suggested that a decrease in corneal neolymphatic vessels was associated with a decrease in the number of macrophages in the cornea (50). This may be because macrophages can secrete paracrine factors and promote lymphatic vessel angiogenesis by binding first to VEGF-C and VEGF-D and then to VEGFR-3 (51). Moreover, M1 macrophages have a greater ability to promote lymphatic vessel formation than M0 and M2 macrophages (52). M1 macrophages can secrete TNF-α, IL-1β, and VEGF-C, thereby promoting lymphatic endothelial cells proliferation (53–55). In summary, macrophages, especially M1 macrophages, can promote CGR by promoting the generation and growth of corneal lymphatic vessels. In this study, the growth of LYVE-1-labeled lymphatic vessels 7 days after surgery was considerably lower in the resveratrol group than in the control group and positively correlated with the growth of corneal neovascularization. This suggests that resveratrol can reduce the generation of new corneal lymphatic vessels after corneal transplantation, thereby preventing CGR. Similar to the above results, a recent study reported that treatment with dimethyl fumarate inhibited M1 macrophage polarization and lymphangiogenesis, prolonging the survival of corneal allografts in rats (56). Moreover, the proportion of DCs in the cervical lymph nodes of rats treated with resveratrol was significantly lower than in those of rats treated with saline. This suggests that a decrease in the generation of corneal lymphatic vessels suppresses the migration of APCs to local lymph nodes.

Our RNA sequencing analysis revealed that allograft rejection was one of the pathways with the greatest DEG enrichment after resveratrol treatment, providing further evidence that resveratrol can act against CGR. Moreover, the analysis also showed that resveratrol can affect T cells in the corneas. This might be due to its effect on macrophages and may also indicate that it can interfere with immune responses after corneal transplantation through various pathways. These results provide possible directions for future research.

In line with the RNA sequencing results, our Simple Western analysis showed that the PI3K, Akt and p-Akt protein contents in the corneas of the resveratrol group were considerably lower than in those of the control group. This suggests that the action of resveratrol against CGR may be related to the PI3K/Akt pathway. The PI3K/Akt signaling pathway, an important intracellular pathway, is related to many biological processes, including cell survival, proliferation, differentiation, and metabolism (57–59). Previous studies have shown that the PI3K/Akt pathway plays a complex and important role in transplant rejection. Regulatory T (Treg) cell therapy is a promising treatment for transplant rejection, and PTEN can promote the optimal function of human Treg cells by downregulating PI3K-Akt activity (60). Akt or PI3K inhibitors have been shown to prolong the survival of heterotopic cardiac allografts in mice (61). However, miR-151-5p upregulation has been shown to activate the PI3K/Akt signaling pathway and regulate the balance between Th17 and Treg cells, possibly preventing CGR (62). Moreover, the PI3K/Akt pathway has also been found to exert different effects on macrophages. The inhibition of the PI3K/Akt-1 pathway has been shown to induce synovial macrophage apoptosis in rheumatoid arthritis (63). Furthermore, it has been reported that different external stimuli can polarize macrophages into M1 or M2 macrophages by regulating the PI3K/Akt pathway (64, 65). These different or even conflicting results may be related to the activation of different subtypes of PI3K or Akt in macrophages (66). Our study provides only preliminary evidence that resveratrol can prevent CGR by regulating the PI3K/Akt pathway. Further research is needed to discover the specific interactions of PI3K/Akt subtypes and the mechanisms underlying their downstream effectors.

Despite the significant findings on the effects and mechanisms of resveratrol on CGR in rats, this study has certain limitations. First, as a preliminary study on drug efficacy, our experimental focus is to explore the impacts of the drug on animals. We confirmed that resveratrol can prevent CGR, and we preliminarily explored the underlying mechanism. In the future, further experiments are needed to explore the mechanisms of action of resveratrol. Second, intraperitoneal resveratrol injection is a simple and direct method selected to help us preliminarily verify the effects of resveratrol on CGR. During the one-month observation period, no significant side effects were observed. However, a longer observation period is required to confirm its safety. Moreover, it would be best to produce resveratrol eye drops. Finally, its efficacy needs to be further verified in trials involving humans before it is considered suitable for clinical use.

In conclusion, our findings suggest that resveratrol can significantly prolong corneal graft survival. Resveratrol can reduce corneal macrophage recruitment and M1 macrophage polarization and decrease pro-inflammatory cytokines and lymphatic vessel generation after corneal transplantation in rats. The PI3K/Akt pathway may be related to the effects of resveratrol on CGR. Resveratrol represents a promising strategy for preventing CGR and delaying its development.

## Data availability statement

The datasets presented in this study can be found in online repositories. The names of the repository/repositories and accession number(s) can be found at: NCBI GSE236626.

## Ethics statement

Ethical approval was not required for the studies on humans in accordance with the local legislation and institutional requirements because only commercially available established cell lines were used. The animal study was approved by the Institutional Animal Care and Use Committee of Zhongshan Ophthalmic Center of Sun Yat-sen University (protocol code O2021044). The study was conducted in accordance with the local legislation and institutional requirements.

## Author contributions

TH, CX, XD, and JW helped in the study design. CX and RG did the major works on animal experiments. MM, CO, and CH helped in animal experiments. CH helped in the statistical analysis. CX took the lead in writing the manuscript, with help from RG. All authors contributed to the article and approved the submitted version.
